# A SARM1-mitochondrial feedback loop drives neuropathogenesis in a Charcot-Marie-Tooth disease type 2A rat model

**DOI:** 10.1172/JCI161566

**Published:** 2022-12-01

**Authors:** Yurie Sato-Yamada, Amy Strickland, Yo Sasaki, Joseph Bloom, Aaron DiAntonio, Jeffrey Milbrandt

**Affiliations:** 1 Department of Genetics, Washington University School of Medicine, St. Louis, Missouri, USA.; 2 Center for Advanced Oral Science, Niigata University Graduate School of Medical and Dental Science, Niigata City, Japan.; 3 Needleman Center for Neurometabolism and Axonal Therapeutics, St. Louis, Missouri, USA.; 4 Department of Developmental Biology and; 5 McDonnell Genome Institute, Washington University School of Medicine, St. Louis, Missouri, USA.

**Keywords:** Neuroscience, Neurodegeneration, Neurological disorders, Neuromuscular disease

## Abstract

Charcot-Marie-Tooth disease type 2A (CMT2A) is an axonal neuropathy caused by mutations in the mitofusin 2 (*MFN2*) gene. *MFN2* mutations result in profound mitochondrial abnormalities, but the mechanism underlying the axonal pathology is unknown. Sterile α and Toll/IL-1 receptor motif–containing 1 (SARM1), the central executioner of axon degeneration, can induce neuropathy and is activated by dysfunctional mitochondria. We tested the role of SARM1 in a rat model carrying a dominant CMT2A mutation (*Mfn2^H361Y^*) that exhibits progressive dying-back axonal degeneration, neuromuscular junction (NMJ) abnormalities, muscle atrophy, and mitochondrial abnormalities — all hallmarks of the human disease. We generated *Sarm1*-KO (*Sarm1^–/–^*) and *Mfn2^H361Y^* *Sarm1* double-mutant rats and found that deletion of *Sarm1* rescued axonal, synaptic, muscle, and functional phenotypes, demonstrating that SARM1 was responsible for much of the neuropathology in this model. Despite the presence of mutant MFN2 protein in these double-mutant rats, loss of SARM1 also dramatically suppressed many mitochondrial defects, including the number, size, and cristae density defects of synaptic mitochondria. This surprising finding indicates that dysfunctional mitochondria activated SARM1 and that activated SARM1 fed back on mitochondria to exacerbate the mitochondrial pathology. As such, this work identifies SARM1 inhibition as a therapeutic candidate for the treatment of CMT2A and other neurodegenerative diseases with prominent mitochondrial pathology.

## Introduction

Charcot-Marie-Tooth disease type 2A (CMT2A) is a common hereditary motor and sensory neuropathy of the peripheral nervous system characterized by progressive, length-dependent axonal degeneration without myelin involvement that predominantly affects the distal limbs. CMT2A tends to have an earlier onset and faster progression than do most CMTs, leaving many patients nonambulatory as children (1, 2). As with all CMTs, there are no disease-modifying treatments. CMT2A is caused by mutations in the mitofusin 2 (*MFN2*) gene. MFN2 is a nucleus-encoded dynamin-like GTPase residing in the outer membrane of mitochondria that plays a critical role in mitochondrial fusion, but also promotes mitochondrial mobility, mitophagy, and interorganelle calcium signaling (3). In patients with CMT2A, neuronal mitochondria have morphological and functional abnormalities (2, 4, 5), but how these mitochondrial defects result in dying-back axon loss is unknown.

Sterile α and Toll/IL-1 receptor motif–containing 1 (SARM1) is the central executioner of the programmed axon destruction pathway (6, 7) and so is a candidate to mediate axon loss in CMT2A. SARM1 is an enzyme that, when activated, cleaves the essential metabolic cofactor NAD^+^ (8), inducing a stereotyped local metabolic collapse with loss of ATP, defects in mitochondrial depolarization and motility defects, followed by calcium influx and ultimately axon fragmentation (9). Loss of SARM1 blocks axon degeneration in mouse models of axotomy (10, 11), traumatic brain injury (12), and glaucoma (13). Notably, loss of SARM1 also preserves axons in multiple models of chemotherapeutic and metabolic peripheral neuropathy (14–18). Nicotinamide mononucleotide adenylyltransferase 2 (NMNAT2) is an NAD^+^ synthesizing enzyme whose loss results in an increase in nicotinamide mononucleotide (NMN) (19) and hence an increase in the NMN/NAD^+^ ratio, enabling NMN to displace NAD^+^ from an allosteric binding site in the autoinhibitory domain of SARM1, triggering SARM1 activation (20). In a mouse model of a human motor neuropathy caused by loss of NMNAT2, SARM1 mediates a slowly progressive motor-predominant neuropathy with axon loss and muscle atrophy (21), both hallmarks of CMT2A. Finally, genetic variants of *SARM1* that encode constitutively active enzymes are enriched in patients with amyotrophic lateral sclerosis (ALS) and other motor neuropathies (22, 23). In addition to the strong evidence that SARM1 induces axon loss in neuropathies, there is also a wealth of data demonstrating that mitochondrial dysfunction activates SARM1 (24–27). Thus, we hypothesized that SARM1 may be activated in CMT2A neurons and induce axon loss. If SARM1 promotes neuropathology in CMT2A, this would open new possibilities for treatment, as both chemical and gene therapy SARM1 inhibitors block axon loss (28–30).

The role of SARM1 in neurodegenerative disease has primarily been tested in the mouse. Although mouse models of CMT2A exist, they have significant limitations (3). Overexpression of human CMT2A–associated *MFN2* variants using different transgenic systems leads to significant phenotypic heterogeneity (31–35), whereas knockin (KI) of a pathogenic mutation into the endogenous mouse *Mfn2* locus does not cause axonal defects (36). To overcome these obstacles in this study, we analyzed the recently described *Mfn2*-KI rat model (37) carrying the strong pathogenic human mutation H361Y (38). This model recapitulates many aspects of human CMT2A (39). We analyzed the neuropathology in this rat model and found a progressive motor axonopathy with neuromuscular junction (NMJ) defects and muscle atrophy. In addition, we observed numerous mitochondrial defects including fewer mitochondria at synapses, abnormal accumulation of aggregated mitochondria near nodes of Ranvier, and mitochondrial shape and cristae density defects that were most prominent in distal portions of the nerve. We generated the *Sarm1*-KO rat as well as the *Mfn2^H361Y^* *Sarm1* double-mutant rat in order to test the role of SARM1 in CMT2A. We demonstrate that SARM1 was required for axon degeneration, NMJ defects, muscle weakness, and hind limb muscle atrophy in the *Mfn2^H361Y^* rat. Therefore, SARM1 was a key driver of neuropathology in this model of CMT2A. Since mutant MFN2 protein is present in the *Mfn2^H361Y^* *Sarm1* rat, we expected that mitochondrial defects would persist. Instead, SARM1 deletion also suppressed defects in mitochondrial localization, size, number, and cristae density. In addition, we observed that deletion of SARM1 improved mitochondrial axon transport in an in vitro assay. These surprising findings demonstrate that dysfunctional mitochondria activated SARM1 to cause the major neuropathological defects in this CMT2A model, and that activated SARM1 fed back onto mitochondria, exacerbating their dysfunction. Hence, SARM1 inhibition is a compelling therapeutic candidate for the treatment of CMT2A and, potentially, the many other neurodegenerative diseases characterized by mitochondrial dysfunction.

## Results

### Mfn2^H361Y/+^ rats recapitulate CMT2A neuromuscular phenotypes.

**Figure 1:**
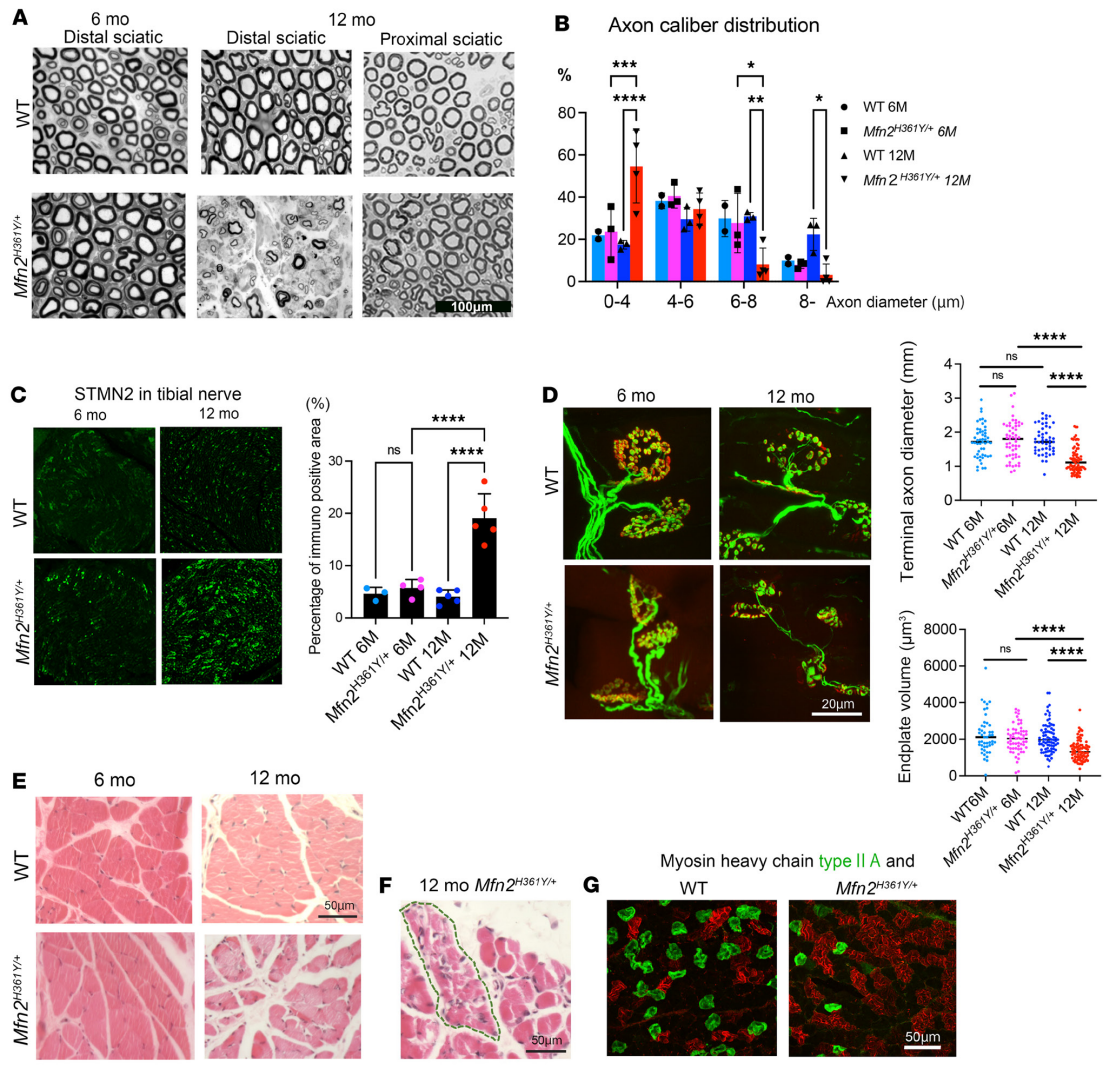
The *Mfn2^H361Y^* mutation causes progressive neurodegeneration and muscle wasting. (**A**) Toluidine blue–stained cross sections of sciatic nerves from WT and *Mfn2^H361Y/+^* rats at 6 and 12 months of age. (**B**) Distribution of axonal diameters of distal sciatic nerves in WT and *Mfn2^H361Y/+^* rats at 6 and 12 months of age (*n* = 3). *P < 0.05, **P < 0.01, ***P < 0.005, and ****P < 0.001, by 1-way ANOVA with Dunnett’s multiple-comparison test. (**C**) STMN2 in tibial nerves of WT and *Mfn2^H361Y/+^* rats at 6 and 12 months of age. Graph shows the percentage of the STMN2 immunopositive area per mm^2^ for each genotype and age (*n* = 3–5). ****P < 0.001, by 1-way ANOVA with Dunnett’s multiple-comparison test. Original magnification, ×100. (**D**) Representative images of NMJs in WT and *Mfn2^H361Y/+^* rats at 6 and 12 months of age, stained in green to detect the marker synaptic vesicle glycoprotein 2A (SV2A) and the axon marker neurofilament medium chain (NEFM) and in red with the postsynaptic endplate marker bungarotoxin. The upper graph exhibits terminal axon diameters and the lower graph exhibits endplate volumes in WT and *Mfn2^H361Y/+^* rats at 6 and 12 months of age (*n* = 3–4). ****P < 0.001, by 1-way ANOVA with Dunnett’s multiple-comparison test. Scale bar: 20 μm. (**E**) Cross sections of H&E-stained lumbrical muscles from WT and *Mfn2^H361Y/+^* rats at 6 and 12 months of age. Scale bar: 50 μm. (**F**) Representative image of group atrophy (encircled by dotted line) in 12-month-old *Mfn2^H361Y/+^* rat muscle stained with H&E. Scale bar: 50 μm. (**G**) Cross sections of gastrocnemius muscle from 12-month-old WT and *Mfn2^H361Y/+^* rats; tissues were immunostained for myosin heavy chain (MHC) type IIA (SC-71) and BF-F3 MHC type IIB to highlight altered fiber type distribution in the mutant animals. Scale bar: 50 μm.

**Figure 2:**
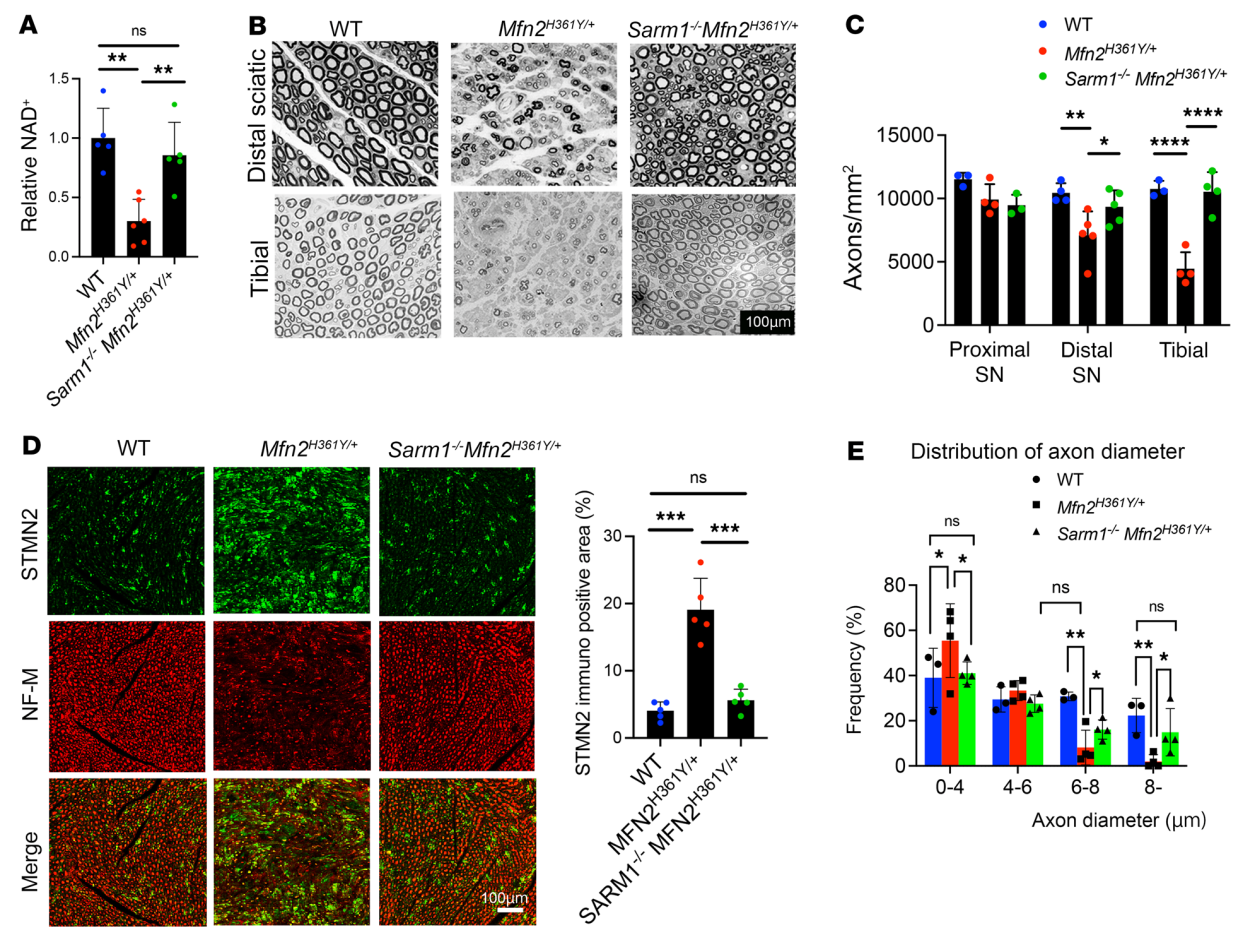
*Sarm1* deletion protects nerves from axonal degeneration in *Mfn2^H361Y/+^* rats. (**A**) Relative NAD^+^ levels in tibial nerves of 12-month-old WT, *Mfn2^H361Y/+^*, and *Sarm1^–/–^Mfn2*^H361Y/+^ rats. Values were normalized to WT (*n* = 3–4). **P < 0.01, by 1-way ANOVA with Dunnett’s multiple-comparison test. (**B**) Toluidine blue–stained cross sections of distal sciatic nerve and tibial nerve from WT, *Mfn2^H361Y/+^*, and *Sarm1^–/–^* *Mfn2^H361Y/+^* rats. Scale bar: 100 μm. (**C**) The number of axons per mm^2^ in proximal sciatic nerve (SN), distal sciatic nerve, and tibial nerve from WT, *Mfn2^H361Y/+^*, and *Sarm1^–/–^* *Mfn2^H361Y/+^* rats (*n* = 3–4). *P < 0.05, **P < 0.01, and ****P < 0.001, by 1-way ANOVA with Dunnett’s multiple-comparison test. (**D**) Immunostaining for STMN2 (green) and NF-M (2H3 antibody, red) in tibial nerve from WT, *Mfn2^H361Y/+^*, and *Sarm1^–/–^* *Mfn2^H361Y/+^* rats. Scale bar: 100 μm. Graph shows the percentage of STMN2-immunopositive area per mm^2^ for each genotype (*n* = 5). ***P < 0.005, by 1-way ANOVA with Dunnett’s multiple-comparison test. (**E**) Distribution of axonal diameters of distal sciatic nerve from WT, *Mfn2^H361Y/+^*, and *Sarm1^–/–^* *Mfn2^H361Y/+^* rats (*n* = 3). There was no significant difference between WT and *Sarm1^–/–^* *Mfn2^H361Y/+^* rats in 0–4, 6–8, and 8 μm axons. *P < 0.05 and **P < 0.01, by 1-way ANOVA with Dunnett’s multiple-comparison test.

Patients with CMT2A have loss of vibratory sense as well as motor weakness that is predominantly in the distal lower limbs and rapidly progresses throughout the first decade of life (1). Sural nerve pathology demonstrates loss of large myelinated fibers with regenerating clusters, but no myelin abnormalities (5). Mitochondrial abnormalities include swelling and dissolution of cristae, as well as aggregation within the axon (4, 39–41). To model this disease, the *Mfn2^H361Y^* mutation, which causes severe early-onset disease in patients, was introduced into the rat genome using CRISPR/Cas9. This model develops progressive functional abnormalities and loss of myelinated axons (37). To investigate the neuronal pathologies in *Mfn2^H361Y/+^* rats, we examined the hind limb nerves and muscles of *Mfn2^H361Y/+^* rats at 6 and 12 months of age. Axons in the sciatic nerve were intact at 6 months of age, but by 12 months, we observed severe axon loss in the distal sciatic nerve (Figure 1A and Figure 2C). Morphometric analysis of axon diameters in 12-month-old *Mfn2^H361Y/+^* rats revealed a decrease in large-caliber axons that was mirrored by an increase in the proportion of small fibers (Figure 1B). This axon size distribution is consistent with a motor neuropathy (42) and is similar to that observed in CMT2A human nerves (39). Patients with CMT2A have symptoms mainly in the lower legs, attributed to more extensive distal axonal degeneration. Tellingly, we observed no axonal defects in proximal sciatic nerves in *Mfn2^H361Y/+^* rats (Figure 1A), consistent with a length-dependent neuropathy. Another pathological signature of CMT2A peripheral nerves is the presence of clusters of regenerating axons along with atrophied axons and onion bulb structures, cardinal evidence of repetitive axonal degeneration and regeneration (39). To detect regenerating axons in distal hind limb nerves, we performed immunostaining for STMN2, a marker of regenerating axons (43). We detected many STMN2^+^ axons in 12-month-old *Mfn2^H361Y/+^* tibial nerves, whereas only a few STMN2-labeled axons were seen in the nerves of 6-month-old *Mfn2^H361Y/+^* or WT rats (Figure 1C).

A number of motor-predominant neurodegenerative diseases involve abnormalities of the structure of the NMJ, an early and important site of neuropathology in type 2 axonal CMT (44). We stained NMJs in lumbrical muscles with antibodies against neurofilament (axon marker) and SV2 (synapse vesicle marker) and α-bungarotoxin (BTX) (postsynaptic marker). Morphological abnormalities found in the NMJs of 12-month-old *Mfn2^H361Y/+^* rats included thin terminal axons and shrunken endplates (Figure 1D). Of note, NMJs innervated by abnormally thin terminal axons have been associated with models of motor neuron disorders including ALS (45) and spinal and bulbar muscular atrophy (46). Lower leg muscle atrophy is a typical clinical symptom of CMT2A (39), so we searched for pathology in the hind paw lumbrical muscles of 12-month old *Mfn2^H361Y/+^* rats. We found smaller muscle fascicles (WT = 435.7 μm^2^ vs. *Mfn2^H361Y/+^* = 356.2 μm^2^, *n* = 3, *P* = 0.045; Figure 1E) in the mutant rats, indicating that the *Mfn2^H361Y^* mutation causes progressive neuropathy in the hind limb. We also observed group atrophy of muscle fibers that was characteristic of denervated muscle (Figure 1F). In chronic neurogenic muscle atrophy, denervated muscle fibers are also reinnervated by neighboring motor axons, which results in enlarged motor units with smaller muscle fibers as well as a change in the distribution of muscle fiber types (i.e., fiber type grouping) (47). Immunostaining with myosin heavy-chain isoform–specific antibodies showed changes in the distribution of muscle fiber types in gastrocnemius muscles of 12-month-old *Mfn2^H361Y^* rats (Figure 1G). The axonal degeneration, NMJ pathology, and muscle atrophy in these *Mfn2^H361Y/+^* rats suggested that they are a faithful model of human CMT2A.

### Sarm1 KO prevents Mfn2^H361Y/+^-associated axon and muscle defects.

SARM1 is an essential component of the programmed axon destruction pathway. Activation of SARM1 triggers axonal degeneration, and the deletion of *SARM1* protects axons from acute injury–induced Wallerian degeneration and toxic and metabolic peripheral neuropathy (10, 11, 14–16, 47). However, to our knowledge, whether SARM1 is involved in progressive neurodegenerative conditions like CMT2A is untested.

To investigate whether SARM1 contributes to axonal degeneration in CMT2A, we generated *Sarm1-*mutant rats using CRISPR gene editing (Supplemental Figure 1A; supplemental material available online with this article; https://doi.org/10.1172/JCI161566DS1). The deletion of *Sarm1* and consequent loss of SARM1 protein were confirmed by DNA sequencing and Western blotting of brain lysates. *Sarm1*-KO rats were further subjected to a sciatic nerve transection assay to test the axon-protective phenotype of this *Sarm1* loss-of-function allele. Plastic sections of distal sciatic nerve from *Sarm1*-KO and WT rats were analyzed 7 days after transection (Supplemental Figure 1D) and showed robust protection of the transected axons, as has been previously reported in *Sarm1*-KO mice (10, 11).

The *Sarm1*-KO rats were then crossed with *Mfn2^H361Y/+^* rats to test whether loss of SARM1 prevents the pathology observed in this CMT2A model. To investigate whether SARM1 is activated in the nerves of *Mfn2^H361Y/+^* rats, we first measured the levels of NAD^+^, the SARM1 substrate (8). When SARM1 is activated, it cleaves NAD^+^, and NAD^+^ levels decrease. Liquid chromatography tandem mass spectrometry (LC-MS/MS) revealed a reduction of NAD expression levels in *Mfn2^H361Y/+^* rat tibial nerves, whereas the levels remained equivalent to WT levels in the nerves of *Sarm1^–/–^* *Mfn2^H361Y/+^* double-mutant rats, consistent with SARM1 activation (Figure 2A). Crucially, the dramatic axonal loss and increased number of regenerating axons (marked by STMN2 staining) observed in *Mfn2^H361Y/+^* nerves were completely abrogated in 12-month-old *Sarm1^–/–^* *Mfn2^H361Y/+^* rats (Figure 2, B–D). The altered distribution of axonal diameters in *Mfn2^H361Y/+^* rat nerves was also suppressed in *Sarm1^–/–^* *Mfn2^H361Y/+^* rats (Figure 2E), indicating the preservation of large-caliber axons with deletion of *Sarm1*.

**Figure 3:**
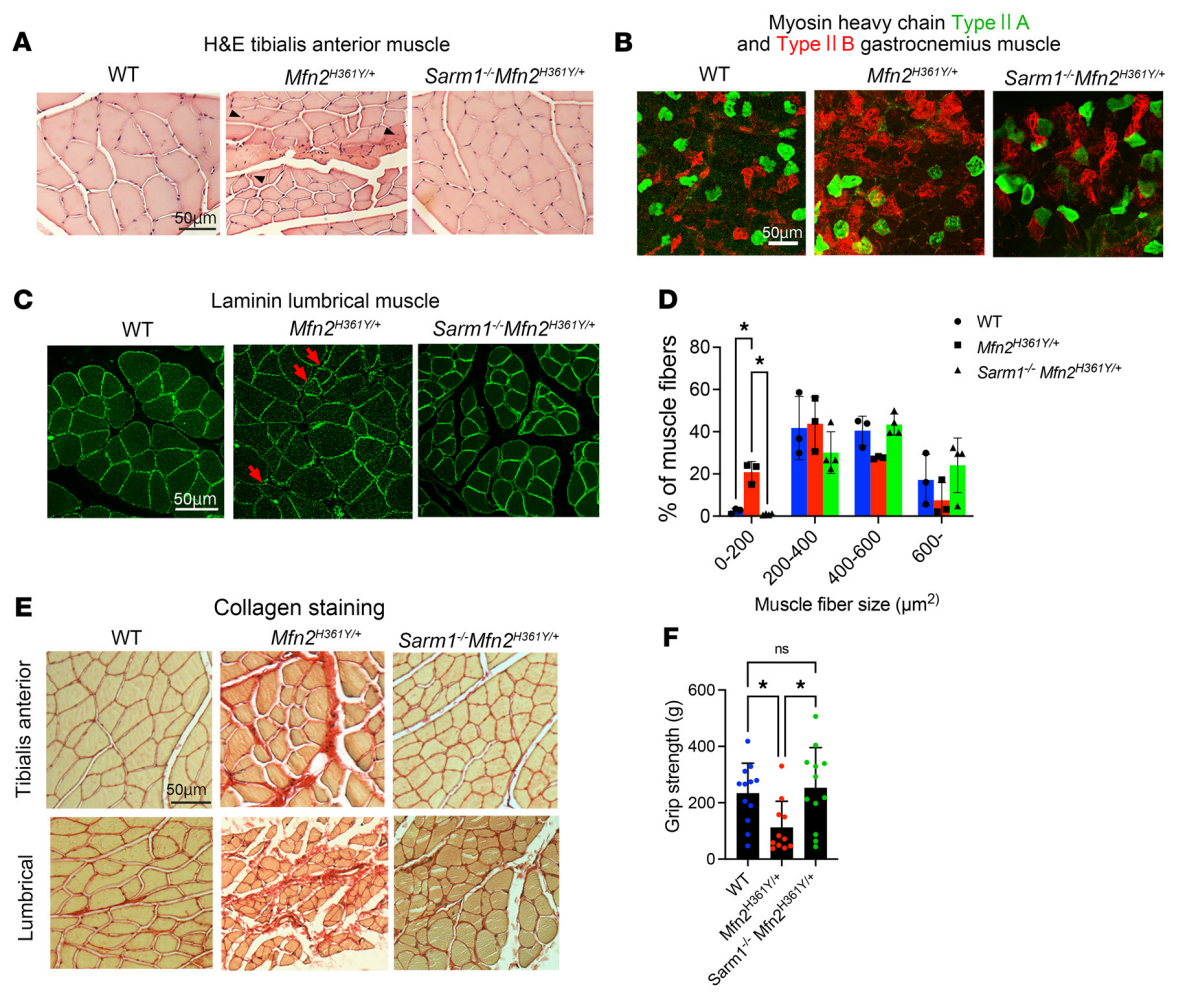
*Sarm1* deletion prevents muscle atrophy in *Mfn2^H361Y/+^* rats. (**A**) Cross sections of H&E-stained tibial anterior muscle from WT, *Mfn2^H361Y/+^*, and *Sarm1^–/–^* *Mfn2^H361Y/+^* rats. Arrowheads indicate nuclei positioned in the center of myofibers, a feature of regenerated muscle. Scale bar: 50 μm. (**B**) Cross sections of gastrocnemius muscle immunostained for MHC type IIA (SC-71 antibody) and IIB (BF-F3 antibody); muscle tissue was from WT, *Mfn2^H361Y/+^*, and *Sarm1^–/–^* *Mfn2^H361Y/+^* rats. Altered distribution of muscle fiber type was observed only in *Mfn2^H361Y/+^* muscle. Scale bar: 50 μm. (**C**) Cross sections of lumbrical muscle immunostained for laminin; muscle tissue was from WT, *Mfn2^H361Y/+^*, and *Sarm1^–/–^* *Mfn2^H361Y/+^* rats. Arrows indicate small atrophied muscle fascicles. Scale bar: 50 μm. (**D**) Distribution of cross-sectional area of tibial anterior muscle fascicles from WT, *Mfn2^H361Y/+^*, and *Sarm1^–/–^* *Mfn2^H361Y/+^* rats (*n* = 3). *P < 0.05, by 1-way ANOVA with Dunnett’s multiple-comparison test. (**E**) Cross sections of Picrosirius red–stained tibial anterior and lumbrical muscle from WT, *Mfn2^H361Y/+^*, and *Sarm1^–/–^* *Mfn2^H361Y/+^* rats. Scale bar: 50 μm. (**F**) Hind limb grip strength measurements performed on 12- to 17-month-old WT, *Mfn2^H361Y/+^*, and *Sarm1^–/–^ Mfn^2H361Y/+^* rats (*n* = 11–12). *P < 0.05, by 1-way ANOVA with Dunnett’s multiple-comparison test.

Along with the axonal deficits, the distal muscles in *Mfn2^H361Y/+^* rats showed severe atrophy (Figure 1, E–G). We further investigated these muscle defects and tested whether *Sarm1* deletion would also improve the loss of muscle integrity caused by the *Mfn2^H361Y/+^* mutation. Group atrophy and abnormal localization of nuclei, which indicate a history of repeated muscle fiber atrophy and regeneration (46–49) (Figure 3A, arrowhead) in *Mfn2^H361Y/+^* rats, was ameliorated in *Sarm1^–/–^* *Mfn2^H361Y/+^* rats (Figure 3A). In addition, the fiber type grouping seen in *Mfn2^H361Y/+^* muscle was not observed in *Sarm1^–/–^* *Mfn2^H361Y/+^* muscle (Figure 3B). Other muscle pathologies associated with motor neuropathy (50) and denervated muscle fibers include changes in muscle fiber diameter (51). We used laminin immunohistochemistry to highlight the muscle fibers and analyzed their cross-sectional area (CSA). We found an increased number of small-caliber muscle fibers (Figure 3C, arrows) and a corresponding decrease in large-caliber muscle fibers in *Mfn2^H361Y/+^* rats (Figure 3D). Muscle denervation is often associated with increased fibrosis due to excessive accumulation of extracellular matrix that replaces functional tissue (52). Picrosirius red staining to detect collagen fibers showed increased collagen fiber content in *Mfn2^H361Y/+^* muscles, with more severe fibrosis and atrophy in lumbrical muscles than in tibialis anterior (TA) muscles, suggesting that the denervation and associated muscle atrophy were more severe at distal than at proximal nerve endings (Figure 3E). In contrast to these results, muscles from *Sarm1^–/–^* *Mfn2^H361Y/+^* rats showed no change in muscle fiber caliber or evidence of fibrosis (Figure 3, C–E). We next investigated whether the preservation of muscle structure was accompanied by maintenance of muscle function. We tested hind limb grip strength in WT, *Mfn2^H361Y/+^*, and *Sarm1^–/–^* *Mfn^2H361Y/+^* rats. The reduction of grip strength in *Mfn2^H361Y/+^* rats was recovered in *Sarm1^–/–^* *Mfn^2H361Y/+^* rats (Figure 3F), demonstrating that loss of SARM1 preserved muscle function. Together, these results are consistent with distal-predominant nerve and muscle pathologies in this CMT2A model. Further, they indicate that the axon degeneration and muscle atrophy due to mitochondrial dysfunction caused by *Mfn2* mutation required SARM1 activity, demonstrating that SARM1 plays a crucial role in chronic, progressive neuropathy in addition to its known roles in acute injury.

### Mfn2^H361Y/+^ NMJ abnormalities are dependent on SARM1 activity.

**Figure 4:**
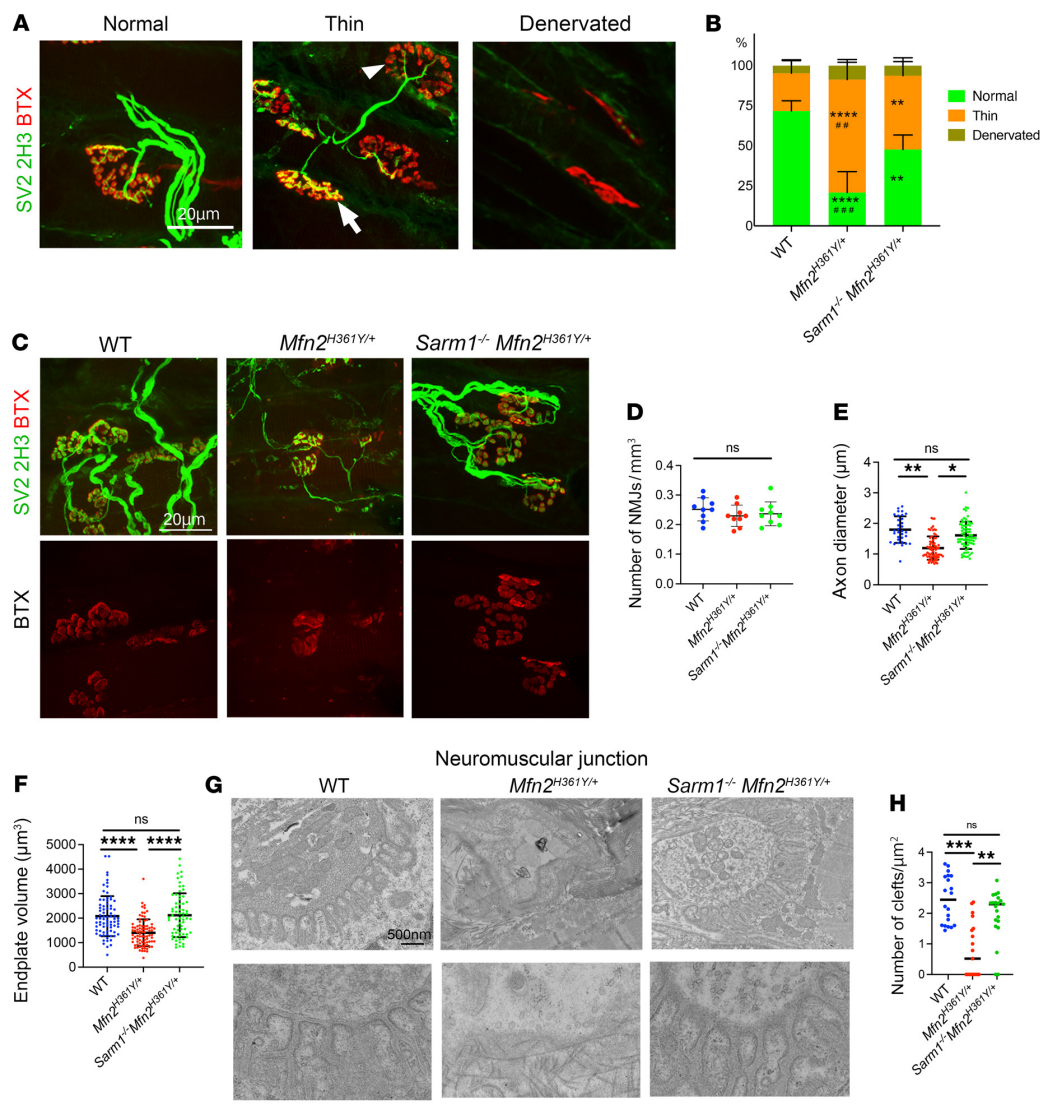
*Sarm1* deletion protects degenerating NMJs in *Mfn2^H361Y/+^* rats. (**A**) Representative images of *Mfn2^H361Y/+^* NMJs with various morphologies stained to detect the synaptic vesicle marker SV2A (SV2), the axon marker NEFM (2H3), and the postsynaptic endplate marker BTX. The arrow indicates an endplate innervated by a thin axon, and the arrowhead indicates an endplate which lacks a presynaptic structure. Scale bar: 20 μm. (**B**) Percentage of each NMJ category in WT, *Mfn2^H361Y/+^*, and *Sarm1^–/–^ Mfn2*^H361Y/+^ lumbrical muscles (*n* = 4). **P < 0.01, and ****P < 0.001, for WT versus *Mfn2^H361Y/+^* or *Sarm1^–/–^ Mfn2*^H361Y/+^; ^##^*P* < 0.01 and ^###^*P* < 0.005, for *Mfn2^H361Y/+^* versus *Sarm1^–/–^ Mfn2*^H361Y/+^; 1-way ANOVA with Dunnett’s multiple-comparison test (**C**) Representative images of NMJs in WT, *Mfn2^H361Y/+^*, and *Sarm1^–/–^ Mfn2*^H361Y/+^ lumbrical muscles. Scale bar: 20 μm. (**D**) Number of NMJs per mm^3^ in WT, *Mfn2^H361Y/+^*, and *Sarm1^–/–^ Mfn2*^H361Y/+^ lumbrical muscles (*n* = 3–4). (**E**) Terminal axon diameters in WT, *Mfn2^H361Y/+^*, and *Sarm1^–/–^ Mfn2*^H361Y/+^ lumbrical muscles (*n* = 3–4). *P < 0.05 and **P < 0.01, by 1-way ANOVA with Dunnett’s multiple-comparison test. (**F**) Endplate volume in WT, *Mfn2^H361Y/+^*, and *Sarm1^–/–^ Mfn2*^H361Y/+^ lumbrical muscle NMJs (*n* = 3–4). ****P < 0.001, by 1-way ANOVA with Dunnett’s multiple-comparison test. Some data in **E** and **F** are repeated from Figure 1D for comparison. (**G**) Electron microscopic images of NMJs in WT, *Mfn2^H361Y/+^*, and *Sarm1^–/–^ Mfn2*^H361Y/+^ lumbrical muscles. Scale bar: 500 nm. (**H**) Number of synaptic clefts per μm^2^ in WT, *Mfn2^H361Y/+^*, and *Sarm1^–/–^ Mfn2*^H361Y/+^ lumbrical muscle NMJs (*n* = 3–4). **P < 0.01 and ***P < 0.005, 1-way ANOVA with Dunnett’s multiple-comparison test.

NMJ morphological abnormalities leading to loss of integrity vary among motor neuron diseases and are correlated with symptom severity (44, 53, 54). To examine the influence of *Sarm1* deletion on mutant *Mfn2*-derived alterations in NMJ morphology, we first classified lumbrical muscle NMJs into the following 3 categories defined previously by others (55, 56) (Figure 4A): (a) “normal NMJs” with typical axonal diameters at terminals (>1.8 μm) and synaptic terminal branches closely opposed to acetylcholine receptor–rich endplates; (b) “thin NMJs” with narrow axonal diameters (*<*1.8 μm) and a mixture of retained (arrow) or eliminated (arrowhead) junctions; and (c) “denervated NMJs” with BTX-labeled postsynaptic sites lacking presynaptic structures or terminal axons. *Mfn2^H361Y/+^* rats had few normal NMJs (*Mfn2^H361Y/+^* 20.8%; WT 71.6%), with most of their NMJs classified as thin (70.5%) and a few as denervated (8.8%) (Figure 4, B and C).

Performing similar analyses of *Sarm1^–/–^* *Mfn^2H361Y/+^* muscles, we found that the endplates and terminal axons looked morphologically normal, with a significantly lower percentage of abnormal NMJs compared with *Mfn2^H361Y/+^* (thin NMJs, 46.1%; denervated NMJs, 6.3%) (Figure 4, B, C, and E). The number of NMJs in *Mfn2^H361Y/+^* muscle was not altered (Figure 4D), however, there was a clear reduction in endplate volume. This reduced endplate volume was completely suppressed by SARM1 loss (Figure 4F), suggesting that the degeneration of muscle fiber segments underlying the synapse was due to muscle fiber denervation (57). Furthermore, ultrastructural analysis of the NMJ revealed severe disorganization with loss of synaptic clefts between pre- and postsynaptic membranes in *Mfn2^H361Y/+^* rats and increased collagen fibers in the presynaptic space, consistent with a loss of normal synaptic innervation. In contrast, there were no ultrastructural abnormalities detected in *Sarm1^–/–^* *Mfn2^H361Y/+^* synapses (Figure 4, G and H). In summary, the *Mfn2^H361Y^* mutation caused NMJ atrophy in a SARM1-dependent manner, suggesting that mitochondrial dysfunction largely led to NMJ and axonal damage via SARM1 activation.

### Mitochondrial defects in Mfn2^H361Y/+^ distal nerves are mitigated by SARM1 deficiency.

**Figure 5:**
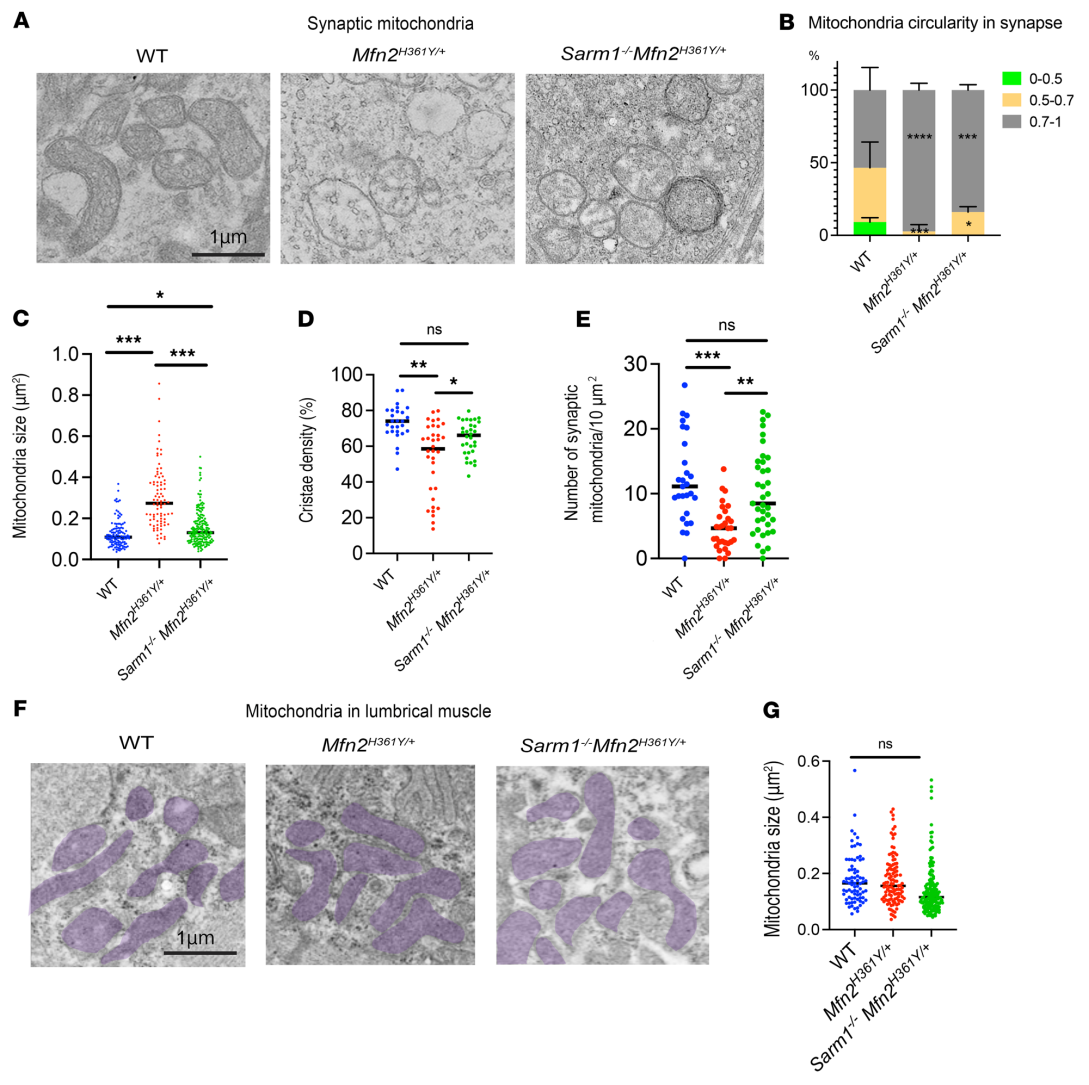
*Sarm1* deletion rescues mitochondrial defects in the synapses of *Mfn2^H361Y/+^* rats. (**A**) Representative images of mitochondria in synapses of WT, *Mfn2^H361Y/+^*, and *Sarm1^–/–^* *Mfn2*^H361Y/+^ rats. Scale bar: 1 μm. (**B**) Percentage of elongated (circularity = 0.7–1), oval (0.5–0.7), and rounded (0–0.5) mitochondria in synapses (*n* = 3). *P < 0.05, ***P < 0.005, and ****P < 0.001, by 1-way ANOVA with Dunnett’s multiple-comparison test. (**C**) Quantification of mitochondria size in synapses (*n* = 3). *P < 0.05 and ***P < 0.005, by 1-way ANOVA with Dunnett’s multiple-comparison test. (**D**) Quantification of cristae density of mitochondria in synapses (*n* = 3). *P < 0.05 and **P < 0.01, by 1-way ANOVA with Dunnett’s multiple-comparison test. (**E**) Quantification of mitochondria density in synapses (*n* = 3). **P < 0.01 and ***P < 0.005, by 1-way ANOVA with Dunnett’s multiple-comparison test. (**F**) Representative images of mitochondria in muscle from WT, *Mfn2^H361Y/+^*, and *Sarm1^–/–^ Mfn2*^H361Y/+^ rats. Scale bar: 1 μm. (**G**) Quantification of mitochondrial size in muscle (*n* = 3).

MFN2 is localized in the mitochondrial outer membrane and is a key player in mitochondrial oxidative function, mitochondrial fusion and transport, ER-mitochondria tethering, and other mitochondrial functions (3). As such, mitochondrial pathology is expected to be associated with *MFN2* mutations, and, indeed, the mitochondria in the nerves of patients with CMT2 display altered morphology and distribution (2). Mitochondria are also actively recruited to synapses to support synaptic activity by maintaining local ATP synthesis (58). Therefore, to examine the effects of *Mfn2* mutation on synaptic mitochondria, we analyzed the number and morphology of mitochondria around the NMJs of lumbrical muscles in *Mfn2^H361Y/+^* rats by electron microscopy. We found a significant reduction in the number of mitochondria in axon termini of *Mfn2^H361Y/+^* rats (Figure 5E). Furthermore, most of the presynaptic mitochondria that were present at these distal synapses displayed a swollen, rounded shape with either a low cristae density or a complete lack of discernible cristae (Figure 5A). Despite the intrinsic mitochondrial defects in these rats, the mitochondria in *Sarm1^–/–^* *Mfn2^H361Y/+^* synapses were present in normal numbers and had largely typical morphology. They were slightly swollen compared with WT synapses (Figure 5, A–C), but cristae density was normal (Figure 5D). We also examined mitochondria in the postsynaptic muscle and observed no abnormalities in the *Mfn2^H361Y/+^* animals (Figure 5, F and G), consistent with observations in samples from patients with CMT2 (2). That SARM1 loss suppressed mitochondrial abnormalities caused by a defective intrinsic mitochondrial protein, MFN2, was surprising and suggests a feedback loop between mitochondrial dysfunction and SARM1 activation triggering a cascade of increasing mitochondrial damage.

**Figure 6:**
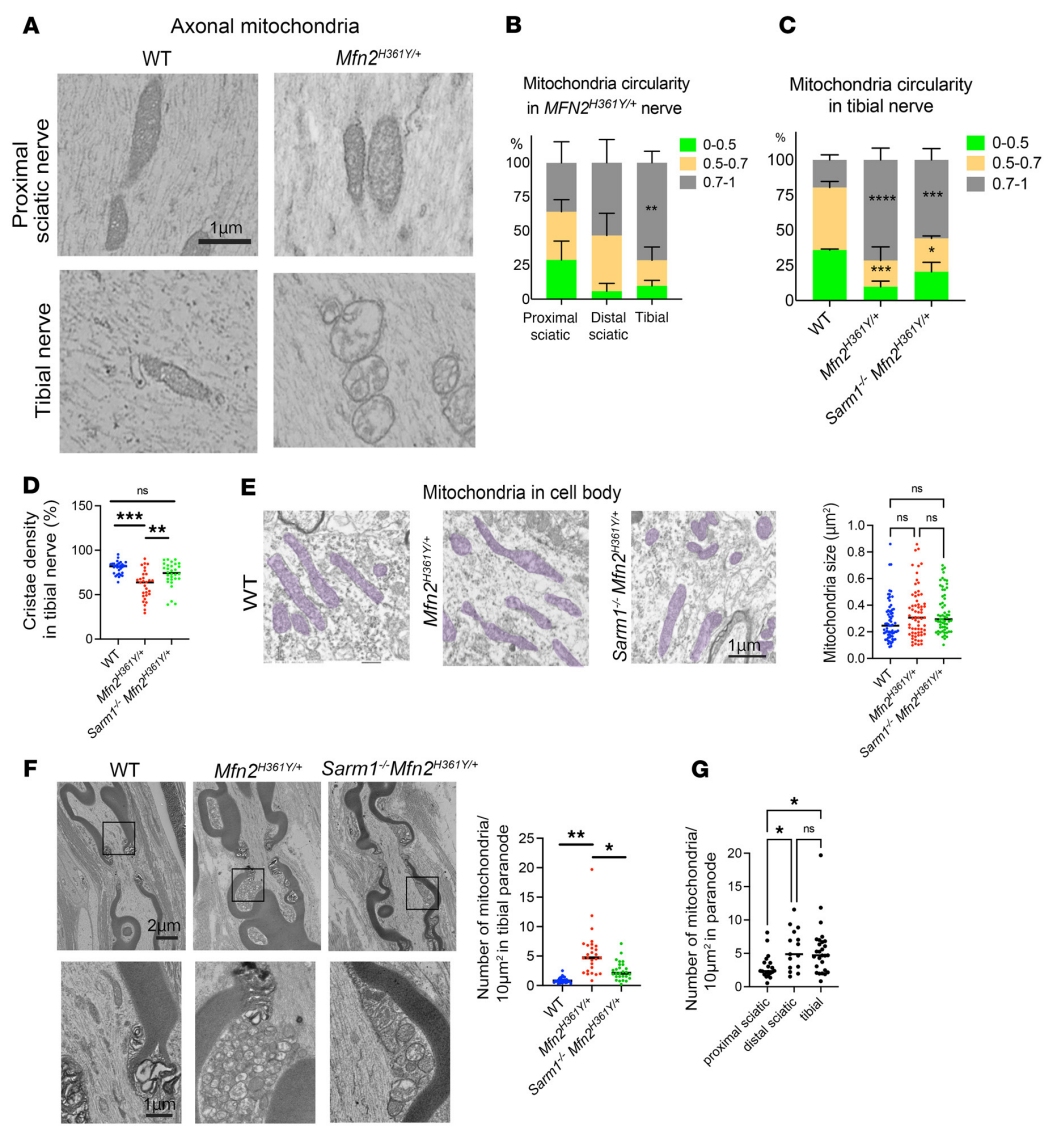
*Sarm1* deletion rescues mitochondrial defects in the axons of *Mfn2^H361Y/+^* rats. (**A**) Electron microscope images of proximal sciatic and tibial nerves from WT and *Mfn2^H361Y/+^* rats showing representative axonal mitochondria. (**B**) Percentage of elongated, oval, and rounded mitochondria categorized by their circularity in three different *Mfn2^H361Y/+^* nerves (*n* = 3). **P < 0.01, comparison between proximal sciatic and tibial; 1-way ANOVA with Dunnett’s multiple-comparison test. (**C**) Percentage of elongated, oval, and rounded mitochondria categorized by their circularity in WT, *Mfn2^H361Y/+^*, and *Sarm1^–/–^ Mfn2*^H361Y/+^ tibial nerve axons (*n* = 3–4). *P < 0.05, ***P < 0.005, and ****P < 0.001, comparison between WT and mutant; 1-way ANOVA with Dunnett’s multiple-comparison test. *Mfn2 ^H361Y/+^* data is repeated from **B** for comparison. (**D**) Cristae density of mitochondria in WT, *Mfn2^H361Y/+^*, and *Sarm1^–/–^ Mfn2*^H361Y/+^ tibial nerve axons (*n* = 3–4). **P < 0.01 and ***P < 0.001, by 1-way ANOVA with Dunnett’s multiple-comparison test. (**E**) Electron microscopic images of mitochondria in WT, *Mfn2^H361Y/+^*, and *Sarm1^–/–^ Mfn2*^H361Y/+^ motor neuron cell bodies (*n* = 3). Scale bar: 1 μm. Graph indicates mitochondrial size in neuronal cell bodies (*n* = 3). (**F**) Representative electron microscopic images of longitudinal axons in tibial nerves showing nodes of Ranvier. Lower images are magnified views of the frames in the upper images showing aggregated mitochondria. Graph represents the number of mitochondria per 10 μm^2^ of paranode in WT, *Mfn2^H361Y/+^*, and *Sarm1^–/–^ Mfn2*^H361Y/+^ axons (*n* = 3). *P < 0.05 and **P < 0.01, by 1-way ANOVA with Dunnett’s multiple-comparison test. (**G**) Number of mitochondria per 10 μm^2^ area of paranode of the proximal sciatic nerve, distal sciatic nerve, and tibial nerve in *Mfn2^H361Y/+^* rats (*n* = 3). *P < 0.05, by 1-way ANOVA with Dunnett’s multiple-comparison test..

Electron microscopic analysis of *Mfn2^H361Y/+^* sciatic and tibial nerve axons also revealed abnormal mitochondria with the same swollen, rounded shape reminiscent of human CMT2A pathology (Figure 6A) (2). Here again, a striking distal preference was apparent in the *Mfn2^H361Y/+^* rat, as the most severe mitochondrial morphological abnormalities were observed at synapses (Figure 5B), followed by distal and then proximal nerve segments (Figure 6, B–D). In contrast, mitochondria in *Mfn2^H361Y/+^* neuronal cell bodies appeared normal (Figure 6E). Thus, the preponderance of mitochondrial deficits occurred in the more distal regions of the nerve including distal axons and, ultimately, at the synapse, consistent with the more severe degeneration of these structures observed in CMT2A neuropathy. The pathological mitochondrial abnormalities were again mitigated by loss of *Sarm1*, with the mitochondrial cristae density in *Sarm1^–/–^* *Mfn^2H361Y/+^* animals being largely normal (Figure 6D). However, the majority of *Sarm1^–/–^* *Mfn^2H361Y/+^* mitochondria still had an aberrantly rounded shape in distal nerves (Figure 6C).

Mitochondria play a central role in energy generation, metabolite synthesis, and calcium buffering (59). Thus, their proper localization in neurons is essential for normal function, and their sparsity in distal regions of the nerve and at the NMJ is likely involved in the pathophysiology of CMT2A. The scarcity of mitochondria in these regions led us to examine their distribution in axons from sciatic and tibial nerves. We found large, abnormal accumulations of mitochondria around the juxtaparanode in *Mfn2^H361Y/+^* axons (Figure 6F), particularly in the distal regions (Figure 6G). Almost all of the mitochondria in these abnormal accumulations had an abnormally rounded shape (Figure 6F). Consistent with the above results, the loss of SARM1 decreased the numbers of mitochondria that accumulated in the juxtaparanodal regions of *Sarm1^–/–^* *Mfn^2H361Y/+^* axons (Figure 6F). The suppression of these mitochondrial abnormalities by loss of SARM1 again suggests an interplay between mitochondrial health and SARM1 axonal energy regulation.

### Mitochondrial transport defect in Mfn2^H361Y/+^ axons is prevented by Sarm1 KO.

**Figure 7:**
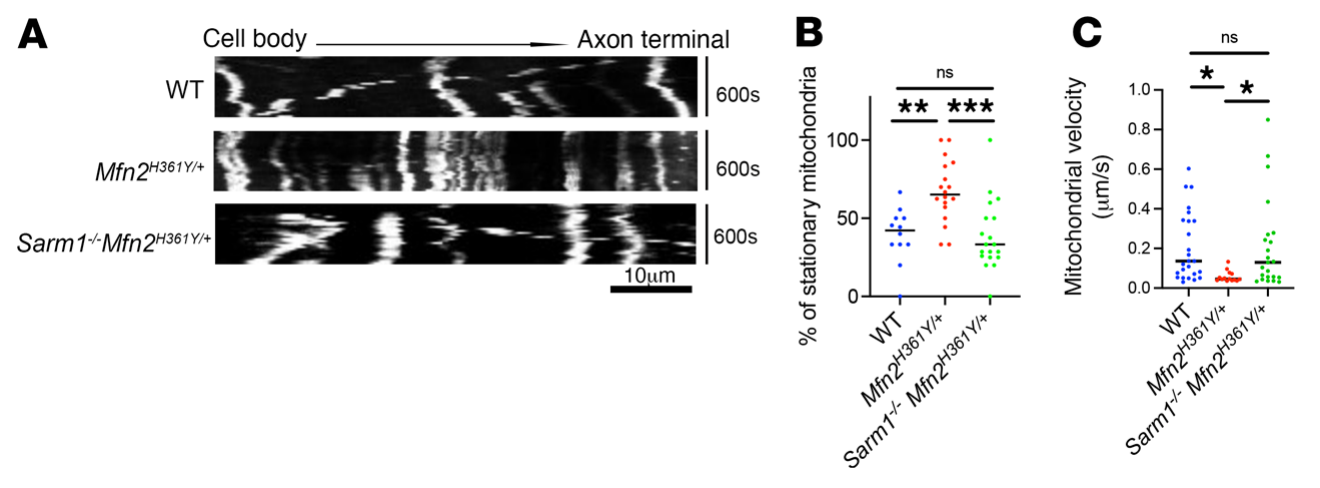
*Sarm1* deletion rescues mitochondria motility in axons of *Mfn2^H361Y/+^* rats. (**A**) Representative kymographs of mitochondrial movement in DRG axons. Scale bar: 10 μm. (**B**) Percentage of stationary mitochondria in WT, *Mfn2^H361Y/+^*, and *Sarm1^–/–^ Mfn2*^H361Y/+^ DRG axons (*n* = 3). **P < 0.01 and ***P < 0.005, by 1-way ANOVA with Dunnett’s multiple-comparison test. (**C**) Motile mitochondrial velocity in WT, *Mfn2^H361Y/+^*, and *Sarm1^–/–^ Mfn2*^H361Y/+^ DRG axons (*n* = 3). *P < 0.05, by 1-way ANOVA with Dunnett’s multiple-comparison test.

MFN2 is necessary for mitochondrial transport in cultured embryonic sensory neuron axons (60), and mitochondrial density was decreased at more distal sites in the *Mfn2^H361Y/+^* rat, suggesting that mitochondrial transport may be affected in these animals. To investigate the effect of the *Mfn2^H361Y^* mutation on mitochondria motility, we infected cultured adult rat dorsal root ganglion (DRG) neurons with Mito-GFP lentivirus to visualize mitochondria in axons. Time-lapse imaging and kymograph analysis demonstrated a significant decrease in mitochondrial motility in *Mfn2^H361Y/+^* axons (Figure 7, A–C), with a significant increase in the percentage of stationary mitochondria and a decreased velocity of the motile mitochondria (Figure 7, A and B). By contrast, the number of stationary mitochondria and mitochondrial motility in *Sarm1^–/–^* *Mfn^2H361Y/+^* axons were similar to what was observed in WT neurons (Figure 7, B and C), indicating that SARM1 activity influences axonal transport and/or the ability of mitochondria to engage the transport machinery. Taken together, these results suggest that SARM1 activity is primarily responsible for the mitochondrial motility defects in axons in the *Mfn2^H361Y^* mutant.

## Discussion

CMT2A is a debilitating axonal neuropathy with prominent axon loss and muscle wasting that is caused by mutations in the mitochondrial fusion protein MFN2. Here, we characterize a recently described rat CMT2A model (37) and demonstrate that it displayed both the hallmark neuropathological features and prominent mitochondrial abnormalities observed in the human disease. SARM1, the central executioner of pathological axon degeneration, is an inducible NAD^+^ hydrolase that can be activated by mitochondrial dysfunction, and we therefore hypothesized that SARM1 mediates the pathology in CMT2A. We generated *Sarm1*-KO rats as well as *Sarm1* *Mfn2* double-mutant rats and found that deletion of *Sarm1* rescued the axonal, synaptic, and muscle structure and function defects in this CMT2A model. Hence, we believe that SARM1 inhibition is an exciting therapeutic candidate for CMT2A. Surprisingly, not only did *Sarm1* KO rescue neuropathological phenotypes, but it also suppressed many of the morphological characteristics of mitochondria associated with mutation of *MFN2*. These findings identify a positive feedback loop whereby dysfunctional mitochondria activate SARM1, which in turn exacerbates mitochondrial dysfunction. We believe this discovery has important implications for the potential efficacy of SARM1 inhibition in the many neurodegenerative diseases with prominent mitochondrial dysfunction. SARM1 inhibition may not only block downstream axon loss, but may also mitigate upstream mitochondrial pathology for which no current treatments exist.

### SARM1 is an essential driver of neuropathology in CMT2A.

CMT2A is a debilitating neuropathy that usually leaves patients nonambulatory as children. It is the most common of the axonal CMT neuropathies and is caused by mutations in the mitochondrial fusion protein *MFN2*. As such, the proximate molecular cause is well understood: loss of MFN2 function disrupts mitochondrial fusion as well as many mitochondrial functions including mitophagy, transport, and interorganelle communication. The fundamental pathological defect in CMT2A is a distal-predominant, dying-back motor and sensory axonopathy with marked muscle atrophy and associated weakness. How do these mitochondrial defects result in dying-back axon loss? Two prominent explanations are that dysfunctional mitochondria cannot meet the axon’s high energy demands, and/or they fail to sufficiently buffer calcium (3, 59). These are both reasonable possibilities, since loss of ATP and calcium influx are 2 important drivers of axon loss. According to these explanations, the distal predominance of the pathology is attributed to defects in mitochondrial transport. However, the identification of SARM1 as the central executioner of axon degeneration in response to pathological insults, including mitochondrial damage, raised an alternate hypothesis — that dysfunctional mitochondria in CMT2A activate SARM1, and that SARM1 activity, rather than mitochondria-autonomous defects, triggers the progressive dying-back axonopathy. Here, we provide strong support for this latter hypothesis.

Our analysis of pathology in the recently described *Mfn2^H361Y^*-KI rat model of CMT2A (37) demonstrated good concordance with the human disease, i.e., progressive distal-predominant axonopathy with muscle atrophy and NMJ defects. Prominent staining for the axon regeneration marker stathmin 2 (STMN2) (43) also provided strong evidence for compensatory axon regeneration in older nerves. To examine the contribution of SARM1 activity to these phenotypes, we used CRISPR-mediated gene editing to generate a *Sarm1*-KO rat and then produced *Mfn2^H361Y^* *Sarm1* double-mutant rats. The absence of SARM1 dramatically suppressed the pathological defects associated with *Mfn2* mutation, rescuing the hallmark axonal, synaptic, and muscle abnormalities. Hence, although compromised mitochondrial function initiates disease, SARM1 is the ultimate driver of neuropathology. If these results hold true in patients, then they have important therapeutic implications, as inhibition of SARM1 would be predicted to dramatically slow disease progression. Both small-molecule and gene therapy SARM1 inhibitors block axon loss in vitro and in vivo (15, 28, 29), and adeno-associated virus–mediated (AAV-mediated) delivery of a *SARM1* dominant-negative transgene ameliorates behavioral and pathological phenotypes in another model of a human motor neuropathy (21).

How might *MFN2* mutations lead to SARM1 activation? While not yet fully understood, a strong hypothesis emerges from the mechanistic details of SARM1 activation (20). The potent SARM1 antagonist and NAD^+^ synthetase NMNAT2 is a highly labile protein whose levels are maintained through continuous transport from neuronal cell bodies to axons (61), such that NMNAT2 levels are highly sensitive to disturbances in axon integrity, transport, or energetics, especially in the most distal portions of long axons. In healthy axons, NMNAT2 suppresses SARM1 activation by locally generating NAD^+^ from its precursor NMN. SARM1 is a metabolic sensor regulated by the competitive binding of NAD^+^ and NMN to an allosteric binding site in its N-terminal autoinhibitory domain. An increase in the NMN/NAD^+^ ratio, as occurs when axonal NMNAT2 levels are low, leads to increased NMN occupancy and induces SARM1 activation (20). Prodegenerative stimuli that activate SARM1 include different types of mitochondrial poisons (24, 25), and these toxins induce the loss of NMNAT2 (26, 27), possibly due to defects in NMNAT2 transport. However, while reduced NMNAT2 transport to distal axons may be a straightforward explanation linking mitochondrial dysfunction and SARM1 activation, we suggest a further, complementary mechanism. Since NMNAT2 requires ATP to synthesize NAD^+^ from NMN, impaired mitochondrial oxidative phosphorylation that impacts local ATP levels could also hinder NAD^+^ synthesis, thus promoting SARM1 activation even in the presence of NMNAT2. These proposed mechanisms are not specific to CMT2A and MFN2 and suggest that SARM1 activation is a common feature of many neurological disorders with mitochondrial dysfunction. If so, SARM1 inhibition may be a broadly applicable therapeutic strategy.

### A mitochondrial-SARM1 feedback loop exacerbates mitochondrial dysfunction in CMT2A.

Loss of SARM1 not only suppressed pathological phenotypes in this CMT2A model, but also prevented many of the mitochondrial defects. This is an unexpected result, as pathogenic MFN2, a mitochondrial outer membrane protein, was still present. If mitochondrial defects were due solely to the loss of MFN2 biochemical function, then those defects should not be suppressed by loss of SARM1. Instead, our findings imply that activated SARM1 feeds back onto mitochondria and exacerbates the mitochondrial phenotypic abnormalities. This idea of a mitochondria-SARM1 feedback loop is consistent with the demonstration that SARM1 is activated by mitochondrial dysfunction (7, 24, 26, 27) and that SARM1 activation causes mitochondrial dysfunction (ref. 9, and reviewed in ref. 62). However, the mechanism by which SARM1 acts upon mitochondria remains undetermined. It is instructive to consider which phenotypes were and were not suppressed by loss of SARM1. In *Mfn2*-mutant neurons, mitochondria take on a much more rounded shape than is seen in WT neurons, consistent with the known roles of mitochondrial fusion as a direct regulator of mitochondrial shape (4). Although there was a modest trend toward improvement of this phenotype with loss of SARM1, the difference was not statistically significant. In contrast, *Sarm1* KO significantly suppressed the decrease in mitochondrial cristae density observed in distal nerves and synapses of *Mfn2*-mutant animals. Cristae density is a morphological defect that correlates with electron transport chain efficacy (63) and is therefore reflective of mitochondrial function. *Sarm1* KO also fully suppresses the increase in the size of mitochondria seen in the *Mfn2* mutant. Mitochondrial swelling occurs when ion homeostasis is impaired in the mitochondrial matrix, disrupting osmotic balance and leading to water influx (63–65). *Sarm1* KO also rescues the large decrease in the number of synaptic mitochondria, which may reflect improvements in both the morphological integrity and axonal transport of mitochondria. For instance, the reduced mitochondrial mobility and velocity observed in axons of cultured adult sensory neurons from *Mfn2^H361Y^* rats are rescued in *Mfn2^H361Y^* *Sarm1* double-mutant neurons. Finally, an additional mitochondrial phenotype was present in the nerves of *Mfn2*-mutant rats — large accumulations of mitochondria in outpouchings adjacent to nodes of Ranvier. While we do not know why mitochondria collected at this location, it is a region of the axon with high energy requirements. This phenotype was also partially suppressed by *Sarm1* KO. Of note, all of these mitochondrial defects became more prominent as one moves distally down the nerve toward the synapse, consistent with the preferential activation of SARM1 in distal axons where short-lived NMNAT2 was first depleted. Taken together, these findings suggest that, while mitochondrial morphology was surely impacted by the direct loss of MFN2 function, all other mitochondrial phenotypes resulted primarily from the actions of SARM1.

How might activated SARM1 disrupt mitochondria? SARM1 is tethered to the outer mitochondrial membrane, and activated SARM1 cleaves NAD^+^, which is essential for mitochondrial function. SARM1 cleaves cytosolic NAD^+^, and mitochondria have the machinery to synthesize their own NAD^+^ via NMNAT3. However, mitochondria also actively import NAD^+^ from the cytosol via the transporter SLC25A51 (66, 67), suggesting that loss of cytosolic NAD^+^ could influence NAD^+^ mitochondrial pools and disrupt NAD^+^-dependent mitochondrial activity including oxidative phosphorylation and ATP generation. In addition, SARM1 activity can lead to rapid ATP loss via inhibition of glycolysis, which depends on adequate NAD^+^ stores. Indeed, the ATP used for fast axonal transport is preferentially generated by glycolysis (68), providing another mechanism for SARM1-dependent mitochondrial transport defects and decreased numbers of synaptic mitochondria in the *Mfn2*-mutant rat. Finally, SARM1 has been linked to mitophagy proteins (69), suggesting it could regulate mitochondrial health in other manners beyond the cleavage of NAD^+^.

The discovery of a mitochondrial dysfunction–SARM1 activation feedback loop has profound implications for understanding the pathogenesis not only of CMT2A, but potentially other forms of CMT2. Many CMT2 disease genes have been identified, and they can be grouped into shared functions (70). Two of these shared functions are mitochondrial homeostasis, as exemplified by mutations in *MFN2* or *OPA1*, and defects in axonal transport, typified by mutations leading to aberrant dynein and kinesin function. As demonstrated here and in previous publications, mitochondrial defects can activate SARM1 (24–27), as can disruptions to axon transport, because NMNAT2 transport is necessary to maintain SARM1 autoinhibition. We suggest that a vicious cycle of mitochondrial dysfunction and SARM1 activation is initiated in CMT2 subtypes associated with disrupted mitochondrial activity or axonal transport, ultimately resulting in SARM1-dependent axon loss and CMT2 pathology. If correct, this hypothesis has 2 important implications. First, SARM1 inhibition may by a disease-modifying therapy for a broad class of CMT2 subtypes. Second, because mitochondrial dysfunction is a feature of many neurodegenerative disorders, this destructive feedback loop may contribute to both the axonal loss and mitochondrial dysfunction in these disparate diseases, with both phenotypes amenable to SARM1-directed therapeutics.

## Methods

### Animals.

As a model of CMT2A, rats which carry the *p.His361Tyr* *Mfn2* mutation (referred to as *Mfn2^H361Y/+^* rats) were generated using CRISPR/Cas9 gene editing technology (37). Functional testing of these *Mfn2^H361Y/+^* rats show abnormalities in gait dynamics at 8 weeks, with a lengthening of the gait cycle by 16 weeks (37). To examine the contribution of SARM1 to CMT2A pathology, generated *Sarm1*-KO rats (referred to here as *Sarm1^–/–^* rats) were generated using the SD1 strain. CRISPR/Cas9 gene editing was used to generate rats with a 110 bp deletion in *Sarm1* exon 2 (Supplemental Figure 1A). This was performed by mixing recombinant Cas9 protein with 2 guide RNAs (gRNAs) (Supplemental Figure 1B) to produce ribonucleoprotein particles that were introduced into rat embryos. To confirm *Sarm1* KO, Western blotting was performed using brain tissue. The SARM1 band was detected at 73 kDa in WT rats and was not present in the *Sarm1^–/–^* rats (Supplemental Figure 1C).

### NMJ staining and analysis.

Rats were transcardially perfused with 20 mL 4% paraformaldehyde (PFA) solution. Dissected lumbrical muscles were postfixed in 4% PFA overnight and then washed with PBS for 15 minutes 3 times. Samples were permeabilized with 2% Triton X-100/PBS (PBST) for 30 minutes and then blocked with 4% BSA in 0.3% PBST for 30 minutes at room temperature. Muscles were incubated with anti-SV2 (Developmental Studies Hybridoma Bank, AB2315387; 1:200) and anti-2H3 (Developmental Studies Hybridoma Bank,; AB2314897;1:100) for 48 hours at 4°C. After incubation with primary antibodies, muscles were incubated with FITC rabbit anti–mouse IgG (Invitrogen, Thermo Fisher Scientific, A21121; 1:400) overnight at 4°C. The samples were then incubated with Alexa Fluor 568–conjugated BTX (Biotium, 00006; 1:500) for 2 hours at room temperature. To analyze NMJ morphology, *Z*-stack images using a confocal microscope (Leica, DFC7000T) were obtained. Maximal intensity projection images were reconstructed, and postsynapse volume and terminal axon diameter were analyzed using Imaris software.

### Immunohistochemistry.

Fixed tissue was processed to make 15 μm thick cryosections as described previously (71). Slides were washed with PBS and blocked with 4% BSA dissolved in 0.3% PBST for 30 minutes at room temperature. The slides were incubated with primary antibodies against STMN2 (43) (1:500), neurofilament-M (2H3; Developmental Studies Hybridoma Bank AB2314897; 1:500), myosin heavy chain type IIA (SC-71, Developmental Studies Hybridoma Bank, AB2147165; 1:500), myosin heavy chain type IIB (BF-F3, Developmental Studies Hybridoma Bank AB2266724; 1:500), or laminin (MilliporeSigma, L9393; 1:1,000) overnight at 4°C. Slides were then washed and incubated with species-appropriate secondary antibodies for 2 hours at room temperature.

### Collagen staining with Picrosirius red.

Paraffin sections (5 μm thick) were prepared as described previously (71), and after deparaffinization, the sections were stained with a Picrosirius Red Kit (VitroVivo Biotech) using the protocol provided. Sections were imaged using a Nikon Eclipse 80i light microscope, and images were analyzed using ImageJ software (NIH).

### Mitochondria and NMJ ultrastructural analysis.

Nerves and lumbrical muscles were processed, and plastic-embedded specimens were prepared as described before (72). Sections (300–400 nm) were collected onto copper grids and then stained with uranyl acetate and lead citrate and imaged by transmission electron microscopy (JEOL1200). The detailed morphology of NMJs and mitochondria was analyzed using ImageJ. Mitochondrial circularity was quantified using the following formula: circularity = 4π × area/perimeter^2,^ as described in a previous report (73). As the value approaches 0.0, it indicates an increasingly elongated polygon. A circularity value of 1.0 indicates a perfect circle.

### Nerve structural analysis by light microscopy.

For light microscope analysis, plastic-embedded specimens were sectioned at 400–600 nm thickness using a Leica EM UC7 Ultramicrotome. Sections were stained with 1% toluidine blue solution. Axons were imaged using a Zeiss Axioscope, and their diameter was analyzed using a IA32 Image Analysis System. To examine the distribution of axonal diameters in nerves, at least 100 axons were measured per rat.

### Western blotting.

Brain lysates were prepared as described before (9). The following antibodies were used: rabbit anti-SARM1 (1:1,000; 13022, Cell Signaling Technology); mouse anti–β-actin (1:4,000; A222, MilliporeSigma); HRP-conjugated anti-rabbit (1:5,000; AB 2307391, Jackson ImmunoResearch); and HRP-conjugated anti-mouse (1:5,000; 115-035-003, Jackson ImmunoResearch).

### Mass spectrometry.

Tibial nerves were homogenized in 160 μL 50% MeOH in water and then centrifuged (15,000*g*, 10 min). Chloroform (50 μL) was added to the supernatant and centrifuged again (15,000*g*, 10 min). The clear aqueous phase was lyophilized, and metabolites were measured as described previously (74).

### DRG cultures.

Ninety-six-well glass-bottomed tissue culture plates (Cellvis, P96-0-N) were coated with poly-d-lysine (0.1 mg/mL; MilliporeSigma) and laminin (3 μg/mL; Invitrogen, Thermo Fisher Scientific) before DRG dissection. DRGs were dissected from adult rats (10–12 months old) (L2–L5) and incubated with 0.5% collagenase (Gibco, Thermo Fisher Scientific, 17104019) at 37°C for 1 hour. Cell suspensions were then triturated by gentle pipetting and washed 3 times with DRG neurobasal growth medium (Gibco, Thermo Fisher Scientific, 21103049) containing 2% B27 (Invitrogen, Thermo Fisher Scientific, 17504044), 100 ng/mL 2.5S NGF (Harlan Bioproducts), 1 μM 5-fluoro-2′-deoxyuridine (MilliporeSigma, 200-072-5), penicillin, and streptomycin. Cell suspensions with densities of 2.0 × 10^5^ cells/mL were prepared and 1.5 μL was placed in the center of each well. Cells were allowed to adhere for 15 minutes, and then 100 μL DRG growth medium was added.

### Mitochondrial motility analysis.

DRG neurons were infected with lentivirus expressing Mito-GFP (1 × 10^3^ to 10 × 10^3^ PFU) at 2 days in vitro (2DIV). To analyze mitochondrial movement, Mito-GFP–labeled mitochondria in distal axons were monitored using the Zeiss Cell Discoverer 7 at 7DIV. Frames were collected every 15 seconds for 10 minutes. Kymographs were generated using the Fiji image processing package in ImageJ, and the percentage of motile mitochondria was quantified.

### Statistics.

GraphPad Prism (GraphPad Software) was used to perform statistical analyses by 1-way ANOVA with Dunnett’s multiple-comparison test. A *P* value of less than 0.05 was considered significant.

### Study approval.

All animal experiments were approved and performed under the direction of institutional animal study guidelines of Washington University in St. Louis (protocol 20-0020).

## Supplementary Material

Supplemental data

## Version 1 10/26/2022

In-Press Preview

## Version 2 12/01/2022

Electronic publication
