# Supplementary material for: A SARM1-mitochondrial feedback loop drives neuropathogenesis in a Charcot-Marie-Tooth disease type 2A rat model
Source: J Clin Invest. 2022 Dec 1;132(23):e161566. doi: 10.1172/JCI161566 (PMC9711878; doi:10.1172/JCI161566)
Supplement: Supplemental data [file jci-132-161566-s038.pdf]

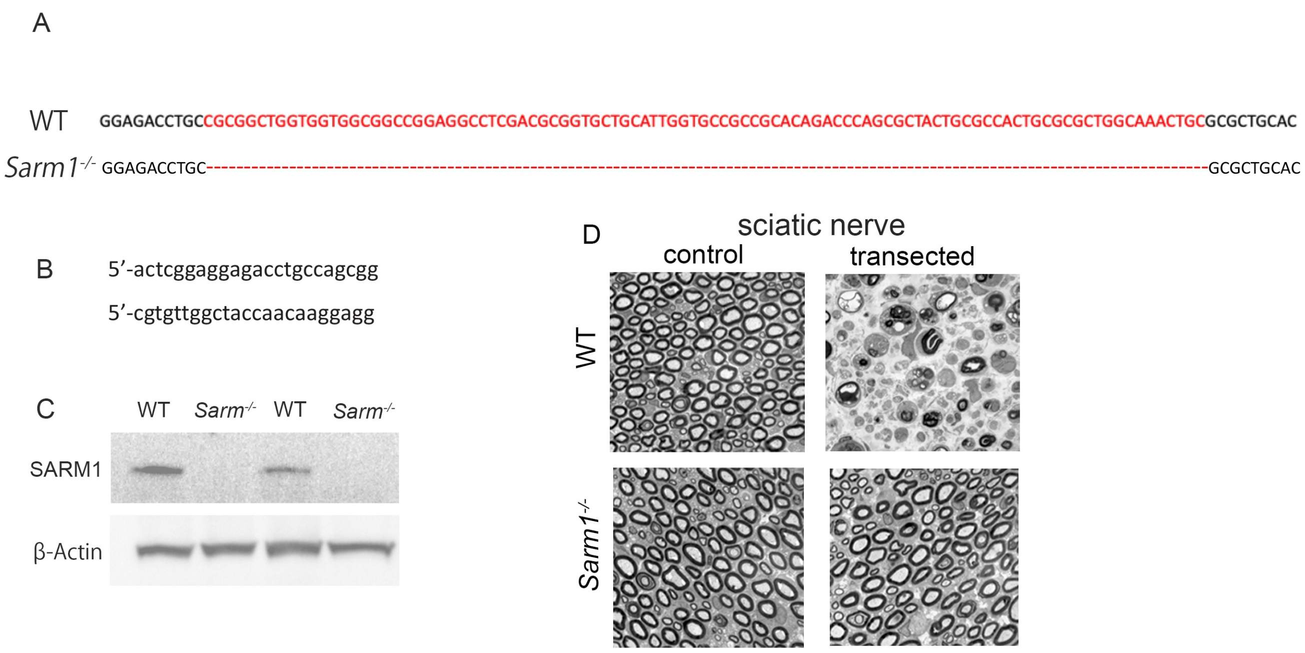

Supplemental figure 1. Generation of Sarm1-/- rats.

(A) The scheme indicates the deletion of Sarm1 exon 2 in Sarm1-/- rats. (B) gRNA sequences used to generate Sarm1-/- rats. (C) Western blot analysis to detect SARM1 expression in the brains of WT and Sarm1-/- rats. (D) Toluidine blue stained cross sections of sciatic nerve from WT and Sarm1-/- rats. Right panels present nerves at 7days after transection, and left panels presents control nerves from the contralateral side.
